# Associations of Pet Ownership with Wheezing and Lung Function in Childhood: Findings from a UK Birth Cohort

**DOI:** 10.1371/journal.pone.0127756

**Published:** 2015-06-10

**Authors:** Simon M. Collin, Raquel Granell, Carri Westgarth, Jane Murray, Elizabeth S. Paul, Jonathan A. C. Sterne, A. John Henderson

**Affiliations:** 1 Centre for Child & Adolescent Health, University of Bristol, Bristol, United Kingdom; 2 School of Social & Community Medicine, University of Bristol, Bristol, United Kingdom; 3 Department of Epidemiology & Population Health, University of Liverpool, Liverpool, United Kingdom; 4 School of Veterinary Science, University of Liverpool, Liverpool, United Kingdom; 5 School of Veterinary Sciences, University of Bristol, Bristol, United Kingdom; National Taiwan University, TAIWAN

## Abstract

**Background:**

Asthma is a heterogeneous condition and differential effects of pet ownership on non-atopic *versus* atopic asthma have been reported. The aim of this study was to investigate whether pet ownership during pregnancy and early childhood was associated with wheezing from birth to age 7 years and with lung function at age 8 years in a UK population-based birth cohort.

**Methods:**

Data from the Avon Longitudinal Study of Parents and Children (ALSPAC) were used to investigate associations of pet ownership at six time-points from pregnancy to age 7 years with concurrent episodes of wheezing, wheezing trajectories (phenotypes) and lung function at age 8 years using logistic regression models adjusted for child’s sex, maternal history of asthma/atopy, maternal smoking during pregnancy, and family adversity.

**Results:**

4,706 children had complete data on pet ownership and wheezing. From birth to age 7 years, cat ownership was associated with an overall 6% lower odds of wheezing (OR=0.94 (0.89-0.99)). Rabbit and rodent ownership was associated with 21% (OR=1.21 (1.12-1.31)) and 11% (OR=1.11 (1.02–1.21)) higher odds of wheezing, respectively, with strongest effects evident during infancy. Rabbit and rodent ownership was positively associated with a ‘persistent wheeze’ phenotype. Pet ownership was not associated with lung function at age 8 years, with the exception of positive associations of rodent and bird ownership with better lung function.

**Conclusions:**

Cat ownership was associated with reduced risk, and rabbit and rodent ownership with increased risk, of wheezing during childhood. The mechanisms behind these differential effects warrant further investigation.

## Introduction

Asthma during childhood has a considerable impact on quality-of-life and healthcare costs [1,2]. A child’s susceptibility to asthma is likely to be determined by interacting genetic and environmental factors [3,4]. Ownership of household pets has attracted attention as an early-life environmental exposure which might play a role in the development of asthma and allergy. Pet ownership is common among households in countries where the incidence and prevalence of childhood asthma and allergies have changed substantially over the past few decades [5,6].

We reported previously that family ownership of domestic pets during early life, including during the pre-natal period, was associated with lower odds of atopic asthma by mid-childhood compared with no pet ownership [7], confirming findings from previous studies [8–10]. Conversely, we also found that pet ownership, particularly of rabbits and rodents, tended to be associated with higher odds of non-atopic asthma. These differential effects of pet ownership probably reflect heterogeneity within childhood asthma, which is recognized to be a complex condition characterised by distinct phenotypes [11], as suggested by analysis of trajectories of wheezing through childhood [12,13]. Differential associations of early-life environmental exposures with wheezing phenotypes, and with phenotypic traits such as lung function, may provide clues as to distinct environmental and pathophysiological processes in asthma causation and exacerbation [14].

Here we used data from the Avon Longitudinal Study of Parents and Children (ALSPAC), a well-characterised UK-based birth cohort, to investigate associations of pet ownership (any pet and specific pet types) at six time-points (from pregnancy to age 7 years) with episodes of wheezing reported at annual intervals from 6 months to 7 years of age, with longitudinal wheezing phenotypes [12], and with lung function (Forced Expiratory Volume in 1 second (FEV_1_) and Forced Vital Capacity (FVC)) measured at age 8 years.

## Methods

### Study population

The Avon Longitudinal Study of Parents and Children (ALSPAC) is a UK population-based study which aims to investigate environmental and genetic influences on the health and development of children [15]. Pregnant women residing in the former Avon Health Authority in south-west England who had an estimated date of delivery between 1 April 1991 and 31 December 1992 were invited to take part, resulting in a cohort of 14,541 pregnancies and 13,978 children alive at 12 months of age (excluding triplets and quads). The primary source of data collection was via self-completion questionnaires sent to mothers at four time-points during pregnancy then at approximately annual intervals following birth. The representative nature of the ALSPAC sample has been investigated by comparison with the 1991 National Census data of mothers with infants under 1 year of age who were residents in the county of Avon. The ALSPAC sample had a slightly greater proportion of mothers who were married or cohabiting, who were owner-occupiers and who had a car in the household. The study had a smaller proportion of ethnic minority mothers. Data used for this submission will be made available on request to the ALSPAC executive committee (alspac-exec@bristol.ac.uk). The ALSPAC data management plan describes in detail the policy regarding data sharing, which is through a system of managed open access (http://www.bristol.ac.uk/alspac/researchers/data-access/documents/alspac-data-management-plan.pdf).

### Ethical approval

Ethical approval for this study was obtained from the ALSPAC Ethics and Law Committee (IRB00003312). Ethical approval for the ‘Focus@8’ research clinics was granted by Weston Local Research Ethics Committee (NHS LREC ref. E177, 21^st^ September 1999). Informed written consent was obtained from the original participants and from the parent(s), next of kin, caretakers, or guardians on behalf of the minors/children enrolled in ALSPAC.

### Outcomes

#### Episodes of wheeze and wheezing phenotypes

Parental reports of child wheezing were obtained from questionnaires sent to the mothers at annual intervals from 6 months to 7 years of age (at approximate ages 6, 18, 32, 42, 54, 69, and 81 months). Presence of wheeze was based on a positive response to the question “In the past 12 months has (your child) had wheezing with whistling on the chest?” or a reported occurrence of wheeze within a list of 15 common symptoms. As previously described, these data were used in a longitudinal latent class analysis to define phenotypes of childhood wheezing, resulting in 6 wheezing phenotypes that were labelled ‘never/infrequent’, ‘transient early’, ‘prolonged early’, ‘intermediate-onset’, ‘late-onset’ and ‘persistent wheezing’ [12].

#### Lung function

Lung function was measured by spirometry according to American Thoracic Society criteria [16] in ‘Focus@8’ research clinics to which all participants were invited at age 8 years (median 103, range 89 to 127 months). Forced expiratory volume in 1 second (FEV_1_) and forced vital capacity (FVC) were converted to units of sex-, age- and height-adjusted standard deviations (z-scores) [17].

### Exposures

#### Pet ownership

Pet ownership questions were asked (in questionnaires about the mother and her environment) during pregnancy (up to 28 weeks gestation) and at child ages of 8 months and 2, 3, 4, and 7 years. The carer of the child (usually the mother) was asked ‘do you have any pets’ and ‘how many of the following pets do you have’. Pet types included cats, dogs, rabbits, rodents (mice, hamster, gerbil etc.), and birds. For the purpose of our study, we defined 4-level categorical variables to indicate whether a cat, dog, rabbit, rodent, bird, or any pet, had been owned/acquired as follows: “Never”; “At age 3 years or later, not before” (owned at age 33 months or at any time thereafter, but not at age 21 months or at any time before, including during pregnancy); “Before and after age 3 years” (owned at any time before age 33 months, including during pregnancy, and at any time thereafter, referred to as ‘continuous’ ownership); “Before but not after 3 years” (owned at any time before, including during pregnancy, but no longer owned at age 33 months or at any time thereafter). We chose age 3 years as a meaningful time point for the acquisition of pets because we wanted to isolate potential reverse-causality in associations of pet ownership with asthma and atopy, specifically, discontinuation of ownership due to the emergence of asthma or atopy during infancy or early childhood [18]. We also defined a binary exposure of “ever” or “never” having owned any pet and specific pet types. Ownership of fish, turtles and tortoises was recorded from age 2 years onwards, and was not coded as a 4-level categorical exposure variable included in the ‘any’ pet ownership binary exposure variable.

### Other variables

#### Potential confounders

A composite ‘family adversity’ index and three factors previously reported to be associated with wheezing phenotypes in ALSPAC (sex of child, maternal history of asthma or allergy, and maternal smoking during pregnancy) [14] were investigated as potential confounders of the association between exposures and outcomes. Maternal history of asthma or allergy was a binary variable derived from responses to an antenatal (18 – 20 weeks gestation) questionnaire in which the mother was asked: a) “Have you ever had any of the following problems?” with responses for “Asthma” of “Yes had it recently” or “Yes in past, not now” coded as ‘yes’; and b) “Would you say that you were allergic to anything?”, with affirmative responses to any of “cat”, “pollen”, “dust”, “insect stings or bites”, or “something else” coded as ‘yes’. Maternal smoking during pregnancy was a binary variable derived from responses to questions put to the mother in an antenatal and a postnatal questionnaire, asking whether the mother had smoked in the first 3 months of the pregnancy, at the mid-point (18 – 20 weeks), or in the last 2 months of the pregnancy.

#### Family Adversity Index

The standard ALSPAC Family Adversity Index (FAI) is derived from responses to questions asked during pregnancy about the following 10 factors, comprising 18 items in total: 1) age of mother at first pregnancy; 2) housing, comprising a) adequacy, b) basic amenities, c) defects, damp, infestation; 3) mother’s and father’s low educational attainment; 4) financial difficulties; 5) relationship with partner, comprising a) status, b) lack of affection, c) cruelty, d) lack of support; 6) family, comprising a) size, b) child in care, not with natural mother, on at-risk register; 7) social network, comprising a) lack of emotional support, b) lack of practical support; 8) substance abuse; 9) crime, comprising a) being in trouble with the police and b) convictions; and 10) psychopathology of the mother (anxiety, depression or suicide attempts). Each of the 18 items is assigned a value of 1 if an adversity is present and 0 if it is not present hence, the FAI has a theoretical range of 0 to 18. FAI scores are calculated where more than half of the items are valid, and non-adversity is assumed for any missing data hence, FAI scores are conservative. Several components of the FAI (age at first pregnancy, overcrowding, family size, rented housing, and maternal anxiety) were previously shown to be associated with wheezing phenotypes [14].

### Statistical methods

Associations of pet ownership with episodes of wheezing at ages 6, 18, 32, 42, and 81 months and with lung function (FEV_1_ and FVC) were investigated using logistic and linear regression models, respectively, adjusted for sex of child, maternal history of asthma or allergy, maternal smoking during pregnancy, and family adversity index. FEV_1_ and FVC were quantified as units of standard deviations (z-scores). The relationship of pet ownership with wheeze over all five time-points was investigated using random effects logit regression models. We analysed ownership of fish, turtles and tortoises in relation to wheezing episodes (as a negative control), but we could not categorize ownership as a 4-level categorical variable because fish/turtle/tortoise ownership was recorded only from age 2 years onwards. Associations of pet ownership with wheezing phenotypes were estimated using multinomial logistic regression models weighted by the probability for each individual of belonging to each phenotype. These probabilities were estimated previously and referred to as the posterior probabilities [12]. According to this method, each child contributes a line of data corresponding to each phenotype for which the child has a non-zero posterior probability. For example, a child might have posterior probability 0.9 of persistent wheeze, 0.1 of transient early wheeze and zero for all other phenotypes. In the regression analyses, this child would contribute two lines of data, the first for persistent wheeze with weight 0.9 and the second for transient early wheeze with weight 0.1. Adjusted relative risk ratios (also known as multinomial odds ratios) were derived in relation to the never/infrequent wheezing phenotype (reference group). Heterogeneity p-values comparing estimated effects across wheezing phenotypes were calculated using Chi-squared tests. Models which used a specific pet type as exposure were not adjusted for the presence of other pets. Pet ownership status over time was modelled by means of the 4-level categorical pet ownership variable, which was coded to indicate non-ownership (i.e. never owned, at any time point), ‘continuous’ ownership (owned at any time point before age 33 months and at any time point thereafter), and ‘discontinuous’ ownership, comprising: pet ‘acquired’ after age 3 years (not previously owned), or pet owned up to age 3 but not afterwards. Estimates for the two ‘discontinuous’ ownership levels give some indication as to whether ‘early’ or ‘late’ ownership has a similar effect on outcomes compared with non-ownership or ‘continuous’ ownership. All analyses were performed using Stata (StataCorp, College Station, TX, USA).

## Results

Of 13,978 children in ALSPAC, 4,706 children (33.7%) had complete data on wheezing, pet ownership and confounders (maternal history of allergy/asthma, maternal smoking during pregnancy and family adversity index). The distributions of exposures and outcomes in the complete data group are summarized in Table 1. Our analysis of lung function was based on 4,177 children (29.9%), and our analyses of pet ownership during pregnancy in relation to wheeze at age 6 months, wheezing phenotypes and lung function were based on 8,661 (62.0%), 5,735 (41.0%) and 5,278 (37.8%) children, respectively. Children with complete data were similar to children with missing data, except that children with complete data were less likely to have had wheeze at age 6 months, had mothers who were less likely to have smoked during pregnancy, and lived in households which had lower levels of adversity and which were less likely to own pets (S1 Table). As reported previously, maternal smoking during pregnancy and family adversity were strongly positively associated with pet ownership, and pet ownership tended to be less common among mothers who reported a history of allergy [7].

**Table 1:** Outcomes and exposures in children with complete data (N = 4,706).

Wheeze at age	6 months	23.2%
	18 months	25.5%
	32 months	21.1%
	42 months	16.7%
	54 months	17.6%
	69 months	14.6%
	81 months	13.2%
Wheezing phenotype*	Never/infrequent	60.2%
	Transient early	16.1%
	Prolonged early	8.6%
	Intermediate onset	2.7%
	Late onset	5.8%
	Persistent	6.7%
Any pet owned/acquired**	Never	15.6%
	Age 3 years or later, not before	19.4%
	Before and after age 3 years	61.3%
	Before but not after 3 years	3.7%
Cat owned/acquired**	Never	57.6%
	Age 3 years or later, not before	7.7%
	Before and after age 3 years	29.6%
	Before but not after 3 years	5.1%
Dog owned/acquired**	Never	69.9%
	Age 3 years or later, not before	7.0%
	Before and after age 3 years	18.4%
	Before but not after 3 years	4.8%
Rabbit owned/acquired**	Never	72.1%
	Age 3 years or later, not before	14.9%
	Before and after age 3 years	8.7%
	Before but not after 3 years	4.4%
Rodent owned/acquired**	Never	65.0%
	Age 3 years or later, not before	25.5%
	Before and after age 3 years	6.8%
	Before but not after 3 years	2.7%
Bird owned/acquired**	Never	86.6%
	Age 3 years or later, not before	4.5%
	Before and after age 3 years	4.9%
	Before but not after 3 years	4.0%

* Frequency distribution weighted by each child’s posterior probability of class membership.

** “Age 3 years or later, not before” = owned at or after age 33 months but not at any time before; “Before and after age 3 years” = owned at any time before age 33 months and at any time thereafter; “Before but not after 3 years” = owned at any time before, but not owned at or at any time after, age 33 months.

### Pet ownership and wheezing

Ownership of any pet was associated with 13% higher odds of wheezing at age 6–8 months (odds ratio (OR) = 1.13; 95% CI 1.02–1.25) but there was no association at any of the later time-points and no overall association. Cat ownership was associated with lower odds of wheezing at ages 18 and 42 months with an overall 6% lower odds of wheezing (OR = 0.94; 95% CI 0.89–0.99) (Table 2, S2 Table). Conversely, owning a rabbit or rodent increased the overall odds of wheezing by 21% (OR = 1.21; 95% CI 1.12–1.31) and 11% (OR = 1.11; 95% CI 1.02–1.21), respectively, with the strongest associations evident during infancy. As expected, ownership of fish, turtles and tortoises was not associated with occurrence of wheezing.

**Table 2:** Associations of pet ownership at different time-points during childhood with concurrent wheezing episodes. *

	Age 6 months	Age 18 months	Age 32 months	Age 42 months	Age 81 months	Overall
Any pet	1.13 (1.02, 1.25)	0.96 (0.87, 1.07)	1.02 (0.91, 1.14)	1.00 (0.88, 1.13)	0.94 (0.78, 1.13)	1.01 (0.96, 1.07)
Cat	0.97 (0.87, 1.09)	0.90 (0.80, 1.01)	1.03 (0.91, 1.16)	0.87 (0.76, 1.00)	0.91 (0.78, 1.07)	0.94 (0.89, 0.99)
Dog	1.11 (0.99, 1.26)	0.91 (0.80, 1.04)	1.01 (0.88, 1.17)	1.03 (0.88, 1.20)	0.90 (0.75, 1.08)	1.00 (0.94, 1.06)
Rabbit	1.38 (1.17, 1.64)	1.07 (0.90, 1.27)	1.22 (1.03, 1.44)	1.23 (1.03, 1.46)	1.14 (0.93, 1.38)	1.21 (1.12, 1.31)
Rodent	1.37 (1.10, 1.71)	1.02 (0.83, 1.27)	1.13 (0.93, 1.37)	1.09 (0.90, 1.32)	1.02 (0.86, 1.21)	1.11 (1.02, 1.21)
Bird	1.13 (0.92, 1.38)	0.92 (0.74, 1.14)	0.99 (0.78, 1.26)	1.15 (0.89, 1.48)	1.15 (0.86, 1.54)	1.04 (0.94, 1.17)
Fish/turtle/tortoise	No data	0.95 (0.82, 1.11)	0.97 (0.84, 1.13)	0.93 (0.79, 1.09)	1.11 (0.94, 1.32)	0.99 (0.91, 1.07)

* Odds ratios (95% CI) from logistic regression models adjusted for sex of child, maternal history of asthma or allergy, maternal smoking during pregnancy, and family adversity index. Pet ownership was ascertained by self-completed questionnaire at 8, 21, 33, 47, and 85 months. Overall estimates for each pet type are from random effects logit regression models across all five time-points.

Pet ownership was not associated with wheezing phenotypes, with the exception of positive associations of rabbit and rodent ownership with persistent wheeze (Table 3, S3 Table). There was no overall heterogeneity in associations across the different phenotypes for any pet type (all P>0.2). Point estimates suggested opposing effects of pet ownership on intermediate onset (inverse association) *versus* persistent wheeze (positive association), and there was statistical evidence of heterogeneity between these two phenotypes for continuous ownership (before and after age 3 years) of any pet type: intermediate onset OR = 0.67 (95% CI 0.42–1.06); persistent wheeze OR = 1.26 (95% CI 0.88–1.80); heterogeneity P = 0.03.

**Table 3:** Associations of pet ownership during childhood with wheezing phenotypes. *

		Transient early	Prolonged early	Intermediate onset	Late onset	Persistent
Any pet	Never	1.00 (ref)	1.00 (ref)	1.00 (ref)	1.00 (ref)	1.00 (ref)
Age 3 years or later, not before		1.11 (0.85, 1.46)	1.17 (0.82, 1.69)	0.76 (0.43, 1.34)	1.10 (0.72, 1.67)	1.21 (0.79, 1.85)
Before and after age 3 years		1.10 (0.87, 1.38)	1.14 (0.84, 1.55)	0.67 (0.42, 1.06)	1.05 (0.74, 1.51)	1.26 (0.88, 1.80)
Before but not after 3 years		1.13 (0.72, 1.80)	1.05 (0.56, 1.97)	1.38 (0.61, 3.14)	1.63 (0.87, 3.05)	1.24 (0.62, 2.47)
Cat	Never	1.00 (ref)	1.00 (ref)	1.00 (ref)	1.00 (ref)	1.00 (ref)
Age 3 years or later, not before		1.13 (0.84, 1.52)	1.09 (0.75, 1.59)	1.27 (0.68, 2.35)	0.95 (0.58, 1.54)	1.09 (0.70, 1.68)
Before and after age 3 years		0.98 (0.82, 1.16)	0.90 (0.72, 1.14)	0.76 (0.50, 1.15)	1.02 (0.77, 1.33)	1.03 (0.80, 1.34)
Before but not after 3 years		0.88 (0.60, 1.28)	0.75 (0.45, 1.25)	1.54 (0.79, 2.99)	1.19 (0.70, 2.01)	1.04 (0.62, 1.74)
Dog	Never	1.00 (ref)	1.00 (ref)	1.00 (ref)	1.00 (ref)	1.00 (ref)
Age 3 years or later, not before		1.16 (0.86, 1.56)	0.96 (0.63, 1.46)	0.71 (0.33, 1.56)	0.99 (0.61, 1.60)	1.46 (0.98, 2.16)
Before and after age 3 years		0.99 (0.81, 1.21)	1.20 (0.93, 1.55)	0.59 (0.34, 1.00)	1.00 (0.73, 1.37)	1.00 (0.74, 1.34)
Before but not after 3 years		1.05 (0.72, 1.52)	1.44 (0.93, 2.22)	1.20 (0.56, 2.55)	0.89 (0.48, 1.65)	0.83 (0.46, 1.49)
Rabbit	Never	1.00 (ref)	1.00 (ref)	1.00 (ref)	1.00 (ref)	1.00 (ref)
Age 3 years or later, not before		1.13 (0.91, 1.40)	1.14 (0.86, 1.52)	0.96 (0.58, 1.59)	0.92 (0.64, 1.32)	1.36 (1.00, 1.86)
Before and after age 3 years		1.07 (0.82, 1.41)	1.27 (0.90, 1.77)	0.69 (0.33, 1.42)	1.06 (0.69, 1.62)	1.28 (0.87, 1.89)
Before but not after 3 years		1.12 (0.75, 1.65)	1.00 (0.59, 1.71)	0.94 (0.37, 2.37)	1.39 (0.79, 2.42)	1.74 (1.07, 2.85)
Rodent	Never	1.00 (ref)	1.00 (ref)	1.00 (ref)	1.00 (ref)	1.00 (ref)
Age 3 years or later, not before		1.07 (0.89, 1.29)	1.17 (0.92, 1.49)	0.80 (0.52, 1.25)	1.04 (0.78, 1.39)	1.31 (1.01, 1.71)
Before and after age 3 years		1.31 (0.98, 1.76)	1.14 (0.76, 1.70)	0.84 (0.40, 1.78)	1.18 (0.74, 1.87)	1.45 (0.96, 2.21)
Before but not after 3 years		0.93 (0.56, 1.53)	1.04 (0.57, 1.92)	1.25 (0.48, 3.28)	0.71 (0.30, 1.69)	1.16 (0.60, 2.25)
Bird	Never	1.00 (ref)	1.00 (ref)	1.00 (ref)	1.00 (ref)	1.00 (ref)
Age 3 years or later, not before		0.83 (0.56, 1.24)	1.00 (0.62, 1.63)	1.36 (0.65, 2.86)	1.23 (0.72, 2.09)	0.75 (0.41, 1.48)
Before and after age 3 years		1.05 (0.75, 1.49)	1.11 (0.72, 1.71)	0.97 (0.43, 2.17)	0.83 (0.46, 1.48)	0.94 (0.56, 1.57)
Before but not after 3 years		1.04 (0.70, 1.54)	0.94 (0.55, 1.59)	0.83 (0.30, 2.26)	0.69 (0.34, 1.43)	0.82 (0.44, 1.52)

* Relative risk ratios (95% CI) from multinomial logistic regression compared with ‘never/infrequent’ wheezing phenotype, adjusted for sex of child, maternal history of asthma or allergy, maternal smoking during pregnancy, and family adversity index.

** “Age 3 years or later, not before” = owned at or after age 33 months but not at any time before; “Before and after age 3 years” = owned at any time before age 33 months and at any time thereafter; “Before but not after 3 years” = owned at any time before, but not owned at or at any time after, age 33 months.

Dog, rabbit and bird ownership during pregnancy were associated with higher odds of wheeze at age 6 months but there were no associations of pet ownership during pregnancy with any of the wheezing phenotypes (Table 4, S2 Table). Of the 57.5% (2,708/4,706) of households recorded as owning any pet type during pregnancy, the majority (87.9% (2,381/2,708)) also reported owning a pet when the child was 6 months old. However, there was some variation by pet type: of households recorded during pregnancy as owning a cat (32.5%), dog (21.4%), rabbit (7.7%), rodent (5.1%) or bird (6.5%), the proportions owning these types of pets when the child was 6 months old were 87.8% (cats), 86.1% (dogs), 68.0% (rabbits), 53.1% (rodents), and 66.7% (birds). Acquisition of a pet in the 6 months after the birth of the child by households which had previously not owned that pet type was uncommon: cats (1.5% (46/3,178)), dogs (1.0% (38/3,701)), rabbits (2.7% (118/4,344)), rodents (2.0% (89/4,467)), and birds (1.3% (56/4,400)).

**Table 4:** Associations of pet ownership during pregnancy with wheeze at 6–8 months and with wheezing phenotypes.

	Odds ratio*	Relative risk ratio (95% CI) compared with ‘never/infrequent’ wheezing phenotype*				
	Wheeze at age 6 months	Transient early	Prolonged early	Intermediate onset	Late onset	Persistent
Any pet	1.09 (0.98, 1.20)	0.99 (0.85, 1.16)	0.98 (0.80, 1.19)	0.86 (0.61, 1.21)	1.04 (0.82, 1.32)	1.01 (0.81, 1.27)
Cat	0.94 (0.84, 1.05)	0.94 (0.80, 1.10)	0.90 (0.73, 1.11)	0.89 (0.61, 1.29)	1.00 (0.78, 1.28)	0.98 (0.78, 1.24)
Dog	1.13 (1.01, 1.27)	1.00 (0.83, 1.20)	1.16 (0.92, 1.45)	0.76 (0.48, 1.20)	0.94 (0.70, 1.25)	0.94 (0.72, 1.23)
Rabbit	1.23 (1.04, 1.45)	1.04 (0.78, 1.37)	1.06 (0.74, 1.52)	0.64 (0.29, 1.40)	0.98 (0.63, 1.52)	1.30 (0.89, 1.89)
Rodent	1.04 (0.84, 1.27)	1.33 (0.97, 1.83)	1.09 (0.60, 2.58)	1.24 (0.60, 2.58)	1.25 (0.77, 2.04)	1.16 (0.73, 1.86)
Bird	1.22 (1.02, 1.46)	1.10 (0.82, 1.48)	1.18 (0.82, 1.70)	1.22 (0.64, 2.34)	0.72 (0.42, 1.22)	0.86 (0.54, 1.36)

* Adjusted for sex of child, maternal history of asthma or allergy, maternal smoking during pregnancy, and family adversity index; pet ownership questionnaire completed from 8^th^ up to 28^th^ week of pregnancy.

### Pet ownership and lung function

Pet ownership was not associated with lung function at age 8 years (Table 5), with the exception of positive associations of rodent and bird ownership with better lung function. For rodent ownership (comparing ever owned *versus* never owned), the mean difference in FEV_1_ z-score was 0.072 (95% CI 0.008 to 0.136, P = 0.03), and the mean difference in FVC z-score was 0.065 (95% CI 0.003 to 0.127, P = 0.04); for bird ownership, the mean difference in FEV_1_ z-score was 0.105 (95% CI 0.014 to 0.195, P = 0.02). There were no consistent patterns in the sign of the point estimate (greater or less than zero) for the mean differences in FEV_1_ and FVC across levels of pet ownership or between pet types.

**Table 5:** Associations of pet ownership with lung function at age 8 years (N = 4,177).

		Mean difference in FEV_1_ *z*-score*	Mean difference in FVC *z*-score*
Any pet**	Age 3 years or later, not before	0.007 (-0.096, 0.110)	-0.019 (-0.120, 0.081)
Before and after age 3 years		0.013 (-0.073, 0.100)	0.006 (-0.078, 0.091)
Before but not after 3 years		-0.063 (-0.240, 0.113)	-0.043 (-0.216, 0.129)
Cat**	Age 3 years or later, not before	0.038 (-0.081, 0.157)	0.028 (-0.089, 0.144)
Before and after age 3 years		0.005 (-0.063, 0.074)	0.010 (-0.057, 0.077)
Before but not after 3 years		-0.018 (-0.161, 0.125)	0.033 (-0.107, 0.173)
Dog**	Age 3 years or later, not before	0.005 (-0.117, 0.127)	-0.026 (-0.146, 0.093)
Before and after age 3 years		0.035 (-0.045, 0.116)	0.029 (-0.049, 0.108)
Before but not after 3 years		-0.065 (-0.212, 0.081)	-0.054 (-0.197, 0.089)
Rabbit**	Age 3 years or later, not before	0.023 (-0.064, 0.110)	0.005 (-0.080, 0.090)
Before and after age 3 years		-0.029 (-0.137, 0.080)	-0.048 (-0.154, 0.058)
Before but not after 3 years		0.036 (-0.116, 0.188)	0.093 (-0.056, 0.241)
Rodent**	Age 3 years or later, not before	0.078 (0.007, 0.148)	0.057 (-0.012, 0.126)
Before and after age 3 years		0.082 (-0.042, 0.206)	0.134 (0.013, 0.256)
Before but not after 3 years		-0.012 (-0.203, 0.178)	-0.026 (-0.212, 0.160)
Bird**	Age 3 years or later, not before	0.127 (-0.018, 0.272)	0.102 (-0.040, 0.244)
Before and after age 3 years		0.009 (-0.137, 0.156)	-0.009 (-0.152, 0.134)
Before but not after 3 years		0.190 (0.032, 0.349)	0.043 (-0.112, 0.198)

* Compared with children from households where pet was never owned, adjusted for sex of child, maternal history of asthma or allergy, maternal smoking during pregnancy, and family adversity index.

** “Age 3 years or later, not before” = owned at or after age 33 months but not at any time before; “Before and after age 3 years” = owned at any time before age 33 months and at any time thereafter; “Before but not after 3 years” = owned at any time before, but not owned at or at any time after, age 33 months.

## Discussion

Rabbit and rodent ownership in this birth cohort was associated with an increased risk, and cat ownership with a slightly reduced risk, of episodes of wheezing during early childhood. The positive associations of rabbit and rodent ownership were most evident during infancy, and also manifested as an increased risk of a ‘persistent wheeze’ phenotype. Cat ownership was not associated with any of the six wheezing phenotypes. We found weak evidence of apparently paradoxical positive associations of rodent and bird ownership with better lung function (FEV_1_ and FVC) at age 8 years, but these are most plausibly ascribed to chance. Dog, rabbit and bird ownership during pregnancy was positively associated with wheezing at age 6 months. These associations may simply reflect the fact that the majority of these households also owned these pet types at age 6 months, rather than indicating specific *in utero* effects.

### Strengths and limitations

ALSPAC is a well-characterized birth cohort which has provided data for several studies into *in utero* and early childhood factors in relation to childhood asthma, wheezing and atopy [7,14,19–23]. A key strength of our study was the availability of exposure measures derived from identical questionnaire items for specific pet types at multiple time-points, from pregnancy through to age 7 years, plus data for potential confounders. The main limitation of our study, as with any birth cohort, relates to losses to follow-up; specifically, the possible effects of higher rates of attrition among children from less affluent families [15]. Given that such children were more likely to be exposed to household pets and to experience wheezing, we would tend to under-estimate associations between pet ownership and wheezing. However, we found that crude associations were affected little by adjustment for family adversity or potential confounding factors such as smoking during pregnancy, and the results of complete-data analyses were similar to results obtained using the maximum amount of available data. This suggests that our results would not be substantially biased by higher losses to follow-up in the lower social strata.

We found that the inclusion of potential confounders made little or no difference to our point estimates, but we cannot discount residual confounding due to unmeasured confounders, for example, number of siblings, time spent outdoors, physical activity, etc. Our dataset did not include factors which might mediate some of our observed associations, in particular whether pets were kept indoors or outdoors, type of dog owned (e.g. moulting *versus* non-moulting), and the frequency and type of contact between children and pets. Future research could investigate dose-response effects in terms of the number and variety of pets owned. We used ownership at any point before age 3 years (including during pregnancy) and at any point thereafter as a pragmatic proxy for ‘continuous’ ownership, rather than strictly identifying households which had uninterrupted pet ownership. We used wheezing phenotypes and pet exposure variables which had been defined for the purpose of previously published analyses—a more sophisticated *de novo* longitudinal analysis using, for example, structural equation modelling, could be used to better elucidate causal associations. This would also minimize the risk of Type 1 error which, because of the large number of permutations of exposure and outcomes, requires us to exercise extra caution in interpreting our results.

### Our findings in the context of other studies

Systematic reviews have reported conflicting evidence, from a wide range of study designs, for the effects of early life exposure to household pets (mainly cats and dogs) and the development of asthma [24–26]. To our knowledge, no other studies have investigated ownership of a wide range of pet types in relation to wheeze at multiple time-points throughout childhood. Brussee *et al* reported inconclusive evidence for (generally positive) associations of cat and dog allergen exposure at 3 months of age with ‘persistent’ and ‘early transient’ wheeze phenotypes, but children were followed up only to age 4 years (and there were few children in the ‘late onset’ phenotype) [27]. Sandin *et al* reported a positive association of dog ownership during the first year of life with a ‘transient’ wheeze phenotype and an inverse association with a ‘late onset’ phenotype, but these phenotypes were also defined for children aged 4 years [28]. In a cohort of children predisposed to asthma or atopy (as indicated by parental history), exposure to cat allergen and dog ownership were inversely associated with repeated wheezing episodes during the first 4–5 years of life [29]. This overall inverse association obscured a positive association, which tended to increase year-on-year, among children whose mothers had a history of asthma [30]. Perzanowski *et al* reported strong positive associations between cat ownership and sensitization to cat allergen by age 2 years, and between sensitization and wheeze at ages 3 and 5 years [31]. However, there was an overall inverse association between cat ownership and risk of wheeze at age 5 years, which was strongest among children who were not sensitized and among children whose mothers did not have a history of asthma. Remes *et al* found an inverse association between dog exposure and onset of wheezing up to age 11 years [32]. This was evident only among children who had a parent with a history of asthma, and there were no associations between cat exposure and wheezing.

Our study contributes an overall inverse association of cat ownership with repeated episodes of wheezing up to the age of 7 years (and no effect of dog ownership) to a diversity of findings for cats and dogs. This diversity can probably be attributed to differences in study design and to variation in factors such as genetic susceptibility (parental history) and background levels of allergen (related to prevalence of pet ownership), and in other environmental and lifestyle factors. Our findings of apparent ‘protective’ effects of cat ownership and ‘harmful’ effects of rabbit and rodent ownership on episodes of wheezing are consistent with our earlier findings from this cohort of children [7]. Specifically, we reported that continuous cat ownership (before and after age 3 years) was associated with 32% (OR = 0.68; 95% CI 0.49–0.95) lower odds of atopic asthma, and continuous ownership of rabbits and rodents with 61% and 86% higher odds, respectively, of non-atopic asthma (OR = 1.61; 95% CI 1.04–2.51 and OR = 1.86; 95% CI 1.15–3.01).

That our other earlier findings of inverse associations of pet ownership with atopic asthma and (weak) positive associations with non-atopic asthma were not clearly evident in our analysis of wheezing phenotypes reflects the non-specific nature of wheeze as a symptom of atopic *versus* non-atopic asthma, both of which were very strongly associated with the intermediate onset and persistent wheezing phenotypes. However, pet ownership tended to be associated with lower risk of the ‘intermediate onset’ wheezing phenotype, which had the strongest association with atopy compared with the never/infrequent phenotype [12]. Similarly, we found evidence of heterogeneity in the effects of ownership of any pet type on ‘intermediate onset’ wheezing (reduced risk) *versus* ‘persistent’ wheeze (increased risk) which echoes our earlier finding of heterogeneity in the effects of ownership of any pet type on atopic asthma (reduced risk) *versus* non-atopic asthma (increased risk) [7], and which appears consistent with the intermediate onset wheezing phenotype being strongly associated with sensitization to common allergens at age 4 years (in two birth cohorts) [33]. In the present study we interpret this apparent heterogeneity with some caution, given the overall absence of heterogeneity across the phenotypes, and the number of possible pairwise tests.

### Implications for clinical practice and future research

Our data point to differential effects across pet types, but do not indicate what aspect(s) of pets and pet ownership may be associated with an increased risk *versus* a protective effect. Pet ownership practices vary by species, and proximity and frequency of pet contact could be important factors, along with factors such as types of bedding used (hay, sawdust, etc). Future research could focus on environmental characteristics associated with different types of pet; fuller details about the domestic husbandry of small mammals such as pet rabbits and rodents will be needed to pinpoint specific risks. Endotoxins, allergens, and other irritants in the home environment might give clues to the causative exposure in much same way as farming studies identified endotoxin as protective exposures for allergy [34–36]. Interactions of environmental characteristics with genetic variation also warrant exploration in relation to exposure to household pets [4,37,38].

### Conclusions

We found ‘protective’ effects of cat ownership and ‘harmful’ effects of rabbit and rodent ownership on wheezing which are consistent with earlier findings from this cohort of children. Parents, or prospective parents, may wish to consider these effects when deciding whether or not to own pets, but whether exposure to pets has a causal effect on the development of wheeze-related disease or whether exposure simply triggers wheezing remains to be elucidated.

## Supporting Information

S1 Table Characteristics of children in our analysis compared with children who had missing data on wheezing and pet ownership. (DOCX)

S2 Table Frequency distributions for pet ownership at different time-points and concurrent wheezing episodes. (DOCX)

S3 Table Distributions of wheezing phenotypes by level of pet ownership. (DOCX)
