# Supplementary material for: Associations of Pet Ownership with Wheezing and Lung Function in Childhood: Findings from a UK Birth Cohort
Source: PLoS One. 2015 Jun 10;10(6):e0127756. doi: 10.1371/journal.pone.0127756 (PMC4465326; doi:10.1371/journal.pone.0127756)
Supplement: S1 Table — (DOCX) [file pone.0127756.s001.docx]

**S1 Table: Characteristics of children in our analysis compared with children who had missing data on wheezing and pet ownership**

|  | | Children with complete data (n=4,706 unless otherwise shown), frequency (%) or mean (SD) | Children with missing data (n=9,272 unless otherwise shown), frequency (%) or mean (SD) | P-value* |
| --- | --- | --- | --- | --- |
| Sex (male) | | 2,429 (51.6%) | 4,791 (51.7%) | 0.95 |
| Maternal history of asthma or allergy (yes) | | 2,220 (47.2%) | 2,834/6,237 (45.4%) | 0.07 |
| Maternal smoking during pregnancy (yes) | | 830 (17.6%) | 2,021/6,353 (31.8%) | <0.001 |
| Family adversity index (range 0 – 10) | | 0.92 (1.28) | 1.43 (1.66), n=5,430 | <0.001 |
| Wheeze at age 6 months | | 1,091 (23.2%) | 1,748/6,193 (28.2%) | <0.001 |
| Wheeze at age 81 months | | 622 (13.2%) | 500/3,676 (13.6%) | 0.61 |
| Any pet owned/acquired** | Never | 725 (15.6%) | 272/4,561 (6.0%) | <0.001 |
| Age 3 years or later, not before | | 911 (19.4%) | 616/4,651 (13.5%) |  |
| Before and after age 3 years | | 2,885 (61.3%) | 3,542/4,651 (77.7%) |  |
| Before but not after 3 years | | 175 (3.7%) | 131/4,651 (2.9%) |  |

* Chi-squared test for proportions, Student’s *t* test for means

** “Age 3 years or later, not before” = owned at or after age 33 months but not at any time before; “Before and after age 3 years” = owned at any time before age 33 months and at any time thereafter; “Before but not after 3 years” = owned at any time before, but not owned at or at any time after, age 33 months
