# Supplementary material for: Associations of Pet Ownership with Wheezing and Lung Function in Childhood: Findings from a UK Birth Cohort
Source: PLoS One. 2015 Jun 10;10(6):e0127756. doi: 10.1371/journal.pone.0127756 (PMC4465326; doi:10.1371/journal.pone.0127756)
Supplement: S2 Table — (DOCX) [file pone.0127756.s002.docx]

**S2 Table: Frequency distributions for pet ownership at different time-points and concurrent wheezing episodes**

|  | Child’s age | Pregnancy | | 6 months | | 18 months | | 32 months | | 42 months | | 81 months | |
| --- | --- | --- | --- | --- | --- | --- | --- | --- | --- | --- | --- | --- | --- |
|  |  | No wheeze (at age 6 months) | Wheeze (at age 6 months) | No wheeze | Wheeze | No wheeze | Wheeze | No wheeze | Wheeze | No wheeze | Wheeze | No wheeze | Wheeze |
| Any pet | Not owned | 1,599 (78.0%) | 439 (22.0%) | 1,701 (78.1%) | 477 (21.9%) | 1,576 (74.8%) | 532 (25.2%) | 1,603 (79.8%) | 406 (20.2%) | 1,574 (83.8%) | 304 (16.2%) | 1,114 (86.4%) | 176 (13.6%) |
|  | Owned | 2,056 (75.9%) | 652 (24.1%) | 1,914 (75.7%) | 614 (24.3%) | 1,929 (74.3%) | 668 (25.7%) | 2,110 (78.3%) | 586 (21.7%) | 2,370 (83.8%) | 457 (16.2%) | 2,970 (86.9%) | 446 (13.1%) |
| Cat | Not owned | 2,441 (76.8%) | 737 (23.2%) | 2,555 (77.0%) | 764 (23.0%) | 2,497 (74.4%) | 859 (25.6%) | 2,674 (79.3%) | 697 (20.7%) | 2,789 (83.4%) | 554 (16.6%) | 2,817 (86.7%) | 434 (13.4%) |
|  | Owned | 1,174 (76.8%) | 354 (23.2%) | 1,060 (76.4%) | 327 (23.6%) | 1,008 (74.7%) | 341 (25.3%) | 1,039 (77.9%) | 295 (22.1%) | 1,155 (84.8%) | 207 (15.2%) | 1,267 (87.1%) | 188 (12.9%) |
| Dog | Not owned | 2,864 (77.4%) | 837 (22.6%) | 2,926 (76.9%) | 877 (23.1%) | 2,874 (74.3%) | 995 (25.7%) | 3,074 (79.3%) | 801 (20.7%) | 3,253 (84.2%) | 612 (15.8%) | 3,236 (86.9%) | 490 (13.2%) |
|  | Owned | 751 (74.7%) | 254 (25.3%) | 689 (76.3%) | 214 (23.7%) | 631 (75.5%) | 205 (24.5%) | 639 (77.0%) | 191 (23.0%) | 691 (82.3%) | 149 (17.7%) | 848 (86.5%) | 132 (13.5%) |
| Rabbit | Not owned | 3,353 (77.2%) | 991 (22.8%) | 3,351 (77.2%) | 991 (22.8%) | 3,211 (74.8%) | 1,082 (25.2%) | 3,340 (79.2%) | 880 (20.9%) | 3,479 (83.9%) | 668 (16.1%) | 3,463 (87.0%) | 516 (13.0%) |
|  | Owned | 262 (72.4%) | 100 (27.6%) | 264 (72.5%) | 100 (27.5%) | 294 (71.4%) | 118 (28.6%) | 373 (76.9%) | 112 (23.1%) | 465 (83.3%) | 93 (16.7%) | 621 (85.4%) | 106 (14.6%) |
| Rodent | Not owned | 3,440 (77.0%) | 1,027 (23.0%) | 3,462 (77.1%) | 1,028 (22.9%) | 3,338 (74.8%) | 1,126 (25.2%) | 3,416 (79.2%) | 897 (20.8%) | 3,505 (84.0%) | 667 (16.0%) | 3,061 (86.8%) | 464 (13.2%) |
|  | Owned | 175 (73.2%) | 64 (26.8%) | 153 (70.8%) | 63 (29.2%) | 167 (69.3%) | 74 (30.7%) | 297 (75.8%) | 95 (24.2%) | 439 (82.4%) | 94 (17.6%) | 1,023 (86.6%) | 158 (13.4%) |
| Bird | Not owned | 3,388 (77.0%) | 1,012 (23.0%) | 3,420 (76.9%) | 1,026 (23.1%) | 3,312 (74.5%) | 1,135 (25.5%) | 3,518 (78.9%) | 939 (21.1%) | 3,730 (83.8%) | 719 (16.2%) | 3,849 (86.9%) | 579 (13.1%) |
|  | Owned | 227 (74.2%) | 79 (25.8%) | 195 (75.0%) | 65 (25.0%) | 193 (75.1%) | 64 (24.9%) | 195 (78.6%) | 53 (21.4%) | 214 (83.6%) | 42 (16.4%) | 235 (84.5%) | 43 (15.5%) |
| Fish/turtle/tortoise | Not owned | No ownership data | | No ownership data | | 3,022 (74.3%) | 1,044 (25.7%) | 3,119 (79.1%) | 824 (20.9%) | 3,207 (83.6%) | 628 (16.4%) | 3,093 (87.2%) | 456 (12.9%) |
|  | Owned | No ownership data | | No ownership data | | 482 (76.0%) | 152 (24.0%) | 591 (77.9%) | 168 (22.1%) | 737 (84.7%) | 133 (15.3%) | 990 (85.6%) | 166 (14.4%) |
