# Supplementary material for: Associations of Pet Ownership with Wheezing and Lung Function in Childhood: Findings from a UK Birth Cohort
Source: PLoS One. 2015 Jun 10;10(6):e0127756. doi: 10.1371/journal.pone.0127756 (PMC4465326; doi:10.1371/journal.pone.0127756)
Supplement: S3 Table — (DOCX) [file pone.0127756.s003.docx]

**S3 Table: Distributions of wheezing phenotypes by level of pet ownership**

|  | |  | Never/ infrequent (%)* | Transient early (%)* | Prolonged early (%)* | Intermediate onset (%)* | Late onset (%)* | Persistent (%)* |
| --- | --- | --- | --- | --- | --- | --- | --- | --- |
| Any pet | | Never | 62.5 | 15.4 | 7.7 | 3.5 | 5.6 | 5.3 |
|  | Age 3 years or later, not before | | 60.2 | 16.5 | 8.7 | 2.7 | 5.9 | 5.9 |
|  | Before and after age 3 years | | 59.8 | 16.2 | 8.8 | 2.4 | 5.6 | 7.3 |
|  | Before but not after 3 years | | 56.8 | 16.0 | 7.7 | 4.6 | 8.3 | 6.6 |
|  |  | |  |  |  |  |  |  |
| Cat | | Never | 60.4 | 16.2 | 8.8 | 2.7 | 5.7 | 6.3 |
|  | Age 3 years or later, not before | | 56.5 | 17.5 | 9.7 | 3.5 | 5.3 | 7.5 |
|  | Before and after age 3 years | | 60.9 | 15.9 | 8.1 | 2.2 | 5.9 | 7.2 |
|  | Before but not after 3 years | | 59.6 | 15.0 | 7.4 | 4.1 | 6.9 | 7.1 |
|  |  | |  |  |  |  |  |  |
| Dog | | Never | 61.0 | 16.0 | 8.0 | 2.9 | 5.8 | 6.3 |
|  | Age 3 years or later, not before | | 56.3 | 18.0 | 8.0 | 2.2 | 5.9 | 9.6 |
|  | Before and after age 3 years | | 59.5 | 15.6 | 10.3 | 1.9 | 5.7 | 7.1 |
|  | Before but not after 3 years | | 56.8 | 16.9 | 11.8 | 3.5 | 5.2 | 5.8 |
|  |  | |  |  |  |  |  |  |
| Rabbit | | Never | 61.0 | 16.0 | 8.4 | 2.8 | 5.7 | 6.0 |
|  | Age 3 years or later, not before | | 58.4 | 16.5 | 9.1 | 2.7 | 5.4 | 8.0 |
|  | Before and after age 3 years | | 58.8 | 16.2 | 9.6 | 1.8 | 5.8 | 7.9 |
|  | Before but not after 3 years | | 54.8 | 16.8 | 8.1 | 2.4 | 7.4 | 10.5 |
|  |  | |  |  |  |  |  |  |
| Rodent | | Never | 61.3 | 15.9 | 8.3 | 2.9 | 5.7 | 6.0 |
|  | Age 3 years or later, not before | | 58.8 | 16.1 | 9.4 | 2.3 | 5.8 | 7.7 |
|  | Before and after age 3 years | | 55.2 | 18.8 | 8.7 | 2.2 | 6.5 | 8.6 |
|  | Before but not after 3 years | | 59.7 | 15.5 | 8.6 | 3.6 | 4.4 | 8.2 |
|  |  | |  |  |  |  |  |  |
| Bird | | Never | 60.1 | 16.2 | 8.6 | 2.7 | 5.8 | 6.8 |
|  | Age 3 years or later, not before | | 61.5 | 13.8 | 8.6 | 3.7 | 7.3 | 5.2 |
|  | Before and after age 3 years | | 58.4 | 17.5 | 9.0 | 2.9 | 5.4 | 6.9 |
|  | Before but not after 3 years | | 61.6 | 16.6 | 8.9 | 2.2 | 4.4 | 6.4 |

* Row percentages weighted by each child’s posterior probability of class membership
